# Pleomorphic Adenoma of the Parotid: Extracapsular Dissection Compared with Superficial Parotidectomy—A 10-Year Retrospective Cohort Study

**DOI:** 10.1155/2014/564053

**Published:** 2014-10-21

**Authors:** Maria Giulia Cristofaro, Eugenia Allegra, Amerigo Giudice, Walter Colangeli, Davide Caruso, Ida Barca, Mario Giudice

**Affiliations:** ^1^Department of Oral and Maxillofacial Surgery, UMG of Catanzaro, Viale Europa, 88100 Catanzaro, Italy; ^2^Department of Otolaryngology, UMG of Catanzaro, Viale Europa, 88100 Catanzaro, Italy; ^3^Department of Odontostomatology, UMG of Catanzaro, Viale Europa, 88100 Catanzaro, Italy

## Abstract

The purpose of this study was to investigate the complication rates and effectiveness of extracapsular dissection compared with superficial parotidectomy for pleomorphic adenomas of the parotid gland from 2002 to 2012. The authors carried out a retrospective cohort study of 198 patients with pleomorphic adenomas of the parotid gland. Extracapsular dissection (ED) or superficial parotidectomy (SP) was performed. The recurrence rate and complications of the two surgical techniques were measured with a univariate analysis of each variable using the appropriate statistical analysis (chi-squared test or *t-*test). A total of 198 patients were enrolled between January 2003 and December 2012. The study included 97 females (48.99%) and 101 males (51.01%) whose mean age was 50.97 years (range 14–75). The type of surgery performed was ED in 153 patients (77.27%, 80 males and 73 females) and SP in 45 patients (22.73%, 21 males and 24 females). The mean follow-up time was 61.02 +/− 4.9 months for the patients treated with ED and 66.4 +/− 4.5 months for the patients treated with SP. Transient facial nerve injury and facial paralysis were significantly more frequent after SP than after ED (*P* = 0.001 and *P* = 0.065, resp.). No significant differences in capsular rupture, recurrence, and salivary fistula were observed after SP or ED: 2.2% versus 3.9%, 2.2% versus 3.3%, and 2.2% versus 0.65%, respectively. Extracapsular dissection may be considered the treatment of choice for pleomorphic adenomas located in the superficial portion of the parotid gland because this technique showed similar effectiveness and fewer side effects than superficial parotidectomy.

## 1. Introduction

Pleomorphic adenomas are the most common benign tumors of the salivary gland, comprising 85% of all salivary gland neoplasms and 60% of the benign tumors of the parotid gland [1–4].

The surgical management of a pleomorphic adenoma has been the subject of controversy for many years, mainly because of the risks of facial nerve injury, capsular rupture, and recurrence [5–8]. A superficial or total parotidectomy involves the resection of a considerable amount of normal parotid tissue with dissection of the facial nerve, causing facial nerve injury and the loss of parotid function [9]. These risks prompted surgeons to adopt less invasive surgical techniques such as extracapsular dissection (ED), which involves the removal of only the tumor and an area of normal parotid parenchyma and the preservation of parotid function, thus minimizing the incidence of facial nerve injury and Frey's syndrome [10–12].

The purpose of this study was to investigate the complication rates and effectiveness of ED and SP for the treatment of pleomorphic adenomas of the parotid gland from 2002 to 2012.

## 2. Materials and Methods

Patients with a pleomorphic adenoma of the parotid gland treated between January 2003 and June 2012 were included in this retrospective cohort study. The diagnosis of a pleomorphic adenoma was established by ultrasonography, head and neck CT or MRI, and FNAC (fine needle aspiration cytology) or FNAB. To be included in the study sample, the patients had a tumor located in the superficial portion of the parotid gland. The mean lesion size was 3.0 +/− 0.5 cm on echography or magnetic or computed tomography. The choice of the surgical technique was randomly assigned.

The study was approved by the appropriate ethics committees, and informed consent was given by the patients.

### 2.1. Superficial Parotidectomy

The skin incision was performed while considering the natural folds of the face and neck flexion (Redon incision), and the incision starts (vertical segment anterior) from the preauricular region and extends up to the ear lobe insertion, reaches the anterior margin of the mastoid, and then continues posteriorly along the mandibular angle.

The superficial muscular aponeurotic system (SMAS) was elevated, and the greater auricular nerve was identified and preserved. The common trunk of the facial nerve was identified, isolated, and controlled by continuous facial nerve monitoring with a neurostimulator (800 Neurosign nerve monitor equipment). After removal of the tumor, hemostasis was ensured using bipolar coagulation, and the facial planes and skin were closed.

### 2.2. Extracapsular Dissection

The skin incision was the same as in the SP. Careful attention was paid to maintaining the integrity of the tumor capsule by performing a wide excision of the parenchyma surrounding the wound (approximately 2-3 mm from the tumor) but without identification of the facial nerve.

In patients with a mass on the angle of the jaw (parotid tail), a modified technique was used. The skin incision of the preauricular region was avoided, and the marginal mandibular branch of the facial nerve was identified and preserved.

### 2.3. Statistical Analysis

The significant difference of the recurrent rates and complications of the two techniques was determined using a univariate analysis of each variable with the appropriate test (chi-squared test or* t*-test). The level of statistical significance was set at *P* = 0.05.

## 3. Results

A total of 198 patients with pleomorphic adenomas of the parotid were included in this study; 97 were females (48.99%) and 101 males (51.01%) with a mean age of 50.97 years (range 14–75). The demographic characteristics of the study population are described in Table 1.

An ED was performed in 153 patients (77.27%, 80 males and 73 females), and an SP was performed in 45 patients (22.73%, 21 males and 24 females). The mean lesion size was 3.0 ± 0.5 cm for ED and 2.5 ± 0.8 for SP treated patients.

The mean follow-up time was 61.02 ± 4.9 months for the patients treated with an ED and 66.4 ± 4.5 months for the patients treated with an SP.

In all patients, the postoperative course was normal without locoregional complications (edema and/or surgical site bleeding) or systemic complications (fever, etc.), and all the patients were discharged after an average of three days from surgery.

Histological examination of the surgical specimen agreed with the FNAB in all patients, confirming the diagnosis of pleomorphic adenoma.

The clinical examination took place on a monthly basis for the first three months, every three months for the first year and every 12 months for another four years for a total follow-up period of five years.

The imaging examinations occurred on a programmed schedule, with ultrasonography every three months for the first year, every six months for the second and third years, and every year for the fourth and fifth years. A CT scan or MRI was scheduled at the first, third, and fifth years. After five years, we determined that there was sufficient evaluation with ultrasonography every 24 months.

The postoperative complications are summarized in Table 2.

In our study, we found that the SP has a higher rate of complications than the ED. A univariate analysis showed a statistically significant association between SP and the occurrence of at least one of the complications examined (*P* = 0.048).

Transient facial nerve injury and facial paralysis were significantly more frequent after SP than after ED (20% versus 4.5%, *P* = 0.001, and 2.2% versus 0%, *P* = 0.065, resp.). The main injuries occurred in the mandibular branch with both techniques.

No significant differences in capsular rupture, recurrence or salivary fistula were observed after SP or ED: 2.2% versus 3.9% (*P* = 0.587), 2.2% versus 3.3%, (*P* = 0.714), and 2.2% versus 0.65% (*P* = 0.355), respectively.

The patients with salivary fistulas were treated by aspiration, a pressure dressing, and a reduced intake of fluid. We had no cases of Frey's syndrome. There were no differences in esthetic satisfaction.

## 4. Discussion and Conclusions

The two main aspects of parotid surgery for benign tumors are the removal of the lesion with adequate margins of healthy parotid tissue surrounding it and preservation of the facial nerve.

Superficial or total parotidectomy has been indicated for pleomorphic adenomas because of the high recurrence rates after enucleation of this benign tumor, which is often related to the incomplete excision or capsule rupture with the dissemination of tumor cells [11, 13].

A superficial or total parotidectomy involves the removal a considerable amount of normal tissue, and fewer branches of the facial nerve are dissected. Other complications are observed more frequently after SP than after ED, including Frey's syndrome, salivary fistula, and injury to the great auricular nerve. The literature data show that ED has similar effectiveness and fewer side effects relative to SP; ED minimizes the incidence of facial nerve paralysis and recurrence and has improved cosmetic results [14–17].

The statistical data collected in this study are in agreement with those reported in the literature and confirm the low morbidity associated with ED. Our incidence of transient facial nerve injury was significantly more frequent after SP than after ED.

The incidence declines from 26% after SP to 11% after ED [1, 12]. This complication does not necessarily result from a branch of nervous neurotmesis. It sometimes results from surgical manipulation that causes a transient nerve injury, and it is proportional to the length of time the nerve is exposed during the surgery. In ED, the facial nerve is not exposed if it is not in contact with the tumor, and when the nerve is in contact with the tumor, only a small portion of the branches of the nerve are handled. The permanent facial nerve damage rate was lower in our data (2.2% after SP versus 0% after ED, *P* = 0.065) than reported in the literature (4% after SP versus 3.5% after ED) (1). In our experience, in a long-term of followup (61.02 ± 4.9 months for the patients treated with ED and 66.4 ± 4.5 months for the patients treated with SP), no significant differences in capsular rupture, recurrence, or salivary fistula were observed after SP or ED: 2.2% versus 3.9% (*P* = 0.587), 2.2% versus 3.3% (*P* = 0.714), and 2.2% versus 0.65% (*P* = 0.355), respectively.

Frey's syndrome, which was not reported in our cases, is more frequent after SP than after ED, and its incidence ranges from 17% after SP to 3% after ED [7, 8, 10, 18]. ED is a microsurgery; in the hands of a novice surgeon or occasional parotid surgeon, it can become a high-risk surgery with various complications [17]. The clinical and statistical data showed a statistically significant association between SP and the occurrence of at least one of the complications examined (*P* = 0.048). For this reason, ED may be considered the treatment of choice for pleomorphic adenomas located in the superficial portion of the parotid gland. We recommend SP for tumors larger than 3.5 cm in diameter, when the lesion is located in the deep portion of the parotid gland or in cases of tumor recurrence. In fact, as Maruyama et al. [19] reported, capsular invasion is more frequent and severe in pleomorphic adenomas larger than 40 mm generally containing more myxoid stroma which promotes vascular invasion. The advantages of ED include the removal of the mass with adequate margins of healthy parotid tissue and a reduction in the side effects after surgery, thus preserving the parotid salivary function. A prolonged followup is recommended [20, 21].

## Figures and Tables

**Table 1:** Demographic characteristics of the study population.

	ED = 153	SP = 45	*P* value
Sex			0.611^*^
Male	80	21	
Female	73	24	
Mean age	58 ± 1.6	51 ± 2.8	**0.063**
Mean lesion size	3.0 ± 0.5	2.5 ± 0.8	**0.451**
Followup	61.02 ± 4.9	66.4 ± 4.5	**0.575**

*P* value according *t*-test and ^*^Fisher's exact test (statistical significance with *P* < 0.05).

**Table 2:** Differences of the recurrent rates and complications of ED versus SP.

Postoperative complications	ED (total = 153)	SP (total = 45)	*P* value
	*n*° (%)	*n*° (%)	
Transient facial nerve injury	7 (4.5%)	9 (20%)	**0.001**
Facial paralysis	0 (0%)	1 (2.2%)	**0.065**
Capsular rupture	6 (6.9%)	1 (2.2%)	**0.587**
Recurrence	5 (3.3%)	1 (2.2%)	**0.714**
Salivary fistula	1 (0.65%)	1 (2.2%)	**0.355**

*P*  value according chi-squared test (statistical significance with *P* < 0.05).
